# Testing for Behavioral and Physiological Responses of Domestic Horses (*Equus caballus*) Across Different Contexts – Consistency Over Time and Effects of Context

**DOI:** 10.3389/fpsyg.2019.00849

**Published:** 2019-04-18

**Authors:** Alexandra Safryghin, Denise V. Hebesberger, Claudia A. F. Wascher

**Affiliations:** Behavioural Ecology Research Group, School of Life Sciences, Anglia Ruskin University, Cambridge, United Kingdom

**Keywords:** individual variation, heart rate, novel object, pre-feeding excitement, domestic horses, *Equus caballus*

## Abstract

In a number of species, consistent behavioral differences between individuals have been described in standardized tests, e.g., novel object, open field test. Different behavioral expressions are reflective of different coping strategies of individuals in stressful situations. A causal link between behavioral responses and the activation of the physiological stress response is assumed but not thoroughly studied. Also, most standard paradigms investigating individual behavioral differences are framed in a fearful context, therefore the present study aimed to add a test in a more positive context, the feeding context. We assessed individual differences in physiological [heart rate (HR)] and behavioral responses (presence or absence of pawing, startle response, defecation, snorting) of 20 domestic horses (*Equus caballus*) in two behavioral experiments, a novel object presentation and a pre-feeding excitement test. Experiments were conducted twice, once between July and August, and once between September and October. Both experiments caused higher mean HR in the first 10 s after stimulus presentation compared to a control condition, but mean HR did not differ between the experimental conditions. In the novel object experiment, horses displaying stress-related behaviors during the experiments also showed a significantly higher HR increase compared to horses which did not display any stress-related behaviors, reflecting a correlation between behavioral and physiological responses to the novel object. On the contrary, in the pre-feeding experiments, horses that showed fewer behavioral responses had a greater HR increase, indicating the physiological response being due to emotional arousal and not behavioral activity. Moreover, HR response to experimental situations varied significantly between individuals. Individual average HR was significantly repeatable across both experiments, whereas HR increase was only significantly repeatable during the novel object and not the pre-feeding experiment. Conversely, behavioral response was not repeatable. In conclusion, our findings show that horses’ behavioral and physiological responses differed between test situations and that emotional reactivity, shown via mean HR and HR increase, is not always displayed behaviorally, suggesting that behavioral and physiological responses may be regulated independently according to context.

## Introduction

Repeatable individual variation of physiological and/or behavioral responses across time and contexts, known as personality or temperament, has been very much in focus of scientific research in recent years and was described in a vast variety of species, including horses (*Equus caballus*) (Goldsmith et al., 1987; Le Scolan et al., 1997; Momozawa et al., 2005; Cockrem, 2007; Lansade et al., 2008; Grajfoner et al., 2010; Olczak et al., 2018). Differences in responses result from an individual’s perception of a potential threat to homeostasis, caused by extrinsic or intrinsic stimuli (stressors) provoking the activation of the physiological stress response in animals and causing behavioral changes (Moberg, 1985; Chrousus and Gold, 1992). In a framework provided by Koolhaas et al. (1999) for rodents, behavioral responses to a stressor usually range from a proactive (fight-flight) to reactive (freeze-hide) axis, linked to low hypothalamic-pituitary-adrenal (HPA) axis and high sympatho-adreno-medullary (SAM) axis reactivity in proactive individuals, and high HPA and low SAM axis reactivity in reactive individuals (Koolhaas et al., 1999). Since then, most studies investigating differences between individuals focus on behavioral responses in experimental situations, such as novel object exposure or open field exploration (Carter et al., 2013). However, the relationship between behavior and individual physiological stress response remains unclear, as it seems to vary across studies.

Individual differences in behavioral and physiological responses to stressors are often determined by early environmental stimuli, such as differences in maternal investment (Stamps, 2003; Claessens et al., 2011). For example, Meaney (2001) showed how adult rats (*Rattus norvegicus*) are more fearful and sensitive to stress if they were raised in the first 8 days of their life by mothers that licked their body and anogenital regions in a frequency lower to the average of the cohort. Contrarily, rats raised by mothers performing a higher-than-average frequency of body and anogenital region licking showed lower fearfulness and sensitivity to stress in adulthood. Moreover, animals can be bred to either show high or low responsiveness to stressors, indicating that individual differences in stress-responsiveness are heritable (Flaherty and Rowan, 1989; Carere et al., 2003). Studies on great tits (*Parus major*) have shown individual physiological stress responses to be related to differences in exploration strategies (Carere and van Oers, 2004) and heritable throughout four generations (Drent et al., 2003).

In horses, diverse factors influencing individual differences in behavior and physiology have been identified in terms of experience, such as habituation (Leiner and Fendt, 2011), diet (Bulmer et al., 2015), handling (Visser et al., 2002), and maternal behavior (Houpt and Hintz, 1983). Similarly, breed has also been found to strongly influence individual reactivity, suggesting a relationship between individual responsiveness and the heritability of traits (Hausberger et al., 2004; Lloyd et al., 2008). Further studies on equine temperament have focused on the assessment of horse responsiveness to different stimuli such as diverse environmental conditions (McCall et al., 2006; Schmidt et al., 2010a,b), novel situations (Visser et al., 2001, 2002; Fureix et al., 2009; Leiner and Fendt, 2011; Ellis et al., 2014) human interactions in terms of both handling (Fureix et al., 2009; König von Brostel et al., 2011; Ellis et al., 2014) and riding (Visser et al., 2008) and have shown how an individual’s response to threat – or “fearfulness” – is stable across time (Visser et al., 2001, 2003; Lansade et al., 2008). However, similar to studies in other species, research investigating personality differences in horses often base their categorization only on observations of behaviors. For example, Grajfoner et al. (2010) compared behavioral ratings between high and low performing horses, showing how the combination of multiple traits, such as a horse being “nice,” “patient,” “easy to handle,” shape the perceived personality of horses. Nonetheless, contrasting results have been found according to the relationship between heart rate (HR) and behavioral parameters. For instance, Momozawa et al. (2003) describe correlations between behavior and HR in their fear-inducing experiments, with more anxious horses showing a higher HR increase and more stress-related behaviors, such as defecation, during the experiment. However, subsequent research reports a lack of this relationship (Christensen et al., 2005; Lansade et al., 2008). Moreover, the lack of stress-related behaviors does not always reflect a lack of physiological stress-response, with studies in horses and cattle (*Bos taurus*) showing that low-behaviorally respondent individuals had higher physiological reactivity (e.g., Jezierski et al., 1999; Welp et al., 2004; Christensen et al., 2005; Lansade et al., 2008).

If a stimulus is perceived as a threat to homeostasis, individuals react with a physiological stress-response, which often reflects an increase in emotional arousal. Emotional arousal is defined as an internal state, which is triggered by specific extrinsic or intrinsic stimuli (Visser et al., 2003; Lansade et al., 2008; Anderson and Adolphs, 2014). Emotional arousal can range between the subject being calm – low arousal, and excited – high arousal, as well as the experience being of positive or negative valence (Russell, 1980). Therefore, the perception of a threatening stimulus can result in an increase in negatively valanced emotional arousal. On the contrary, if it is not perceived as threat but as positively exciting, such stimulus would cause a positively-valanced emotional arousal. The activation of a physiological stress response is often quantified by measuring HR, which therefore can be regarded as a valid standardized, objective, and non-invasive indicator of emotional arousal. Emotional arousal causes changes in behavior, cognition, and physiology (Anderson and Adolphs, 2014). Studies in non-human animals mostly use behavioral measures to quantify emotional arousal (e.g., Anderson et al., 2015; Barnard et al., 2015; Finlayson et al., 2016; Bennett et al., 2017; Albuquerque et al., 2018). Briefer et al. (2015) have studied the effect of emotional arousal and valence on the physiological and behavioral response in goats, showing both, positive (feeding) as well as negative (frustration, isolation) emotional context to cause a significant HR increase and changes in behavior like ear posture compared to a control situation. However, emotional arousal was also found of not being always expressed behaviorally (Wascher et al., 2008). Therefore, it is important to understand how behavior, emotional, and physiological responses are linked.

In the present study, we aim at investigating individual differences in emotional arousal in horses in response to two experimental paradigms, a novel object exposure and a test of pre-feeding excitement. Experimental assessments of animal personality usually focus on stressful contexts, e.g., novel object exposure, open field test. In this study, we aim to investigate consistencies in behavioral and physiological responses across contexts of different valence. Furthermore, we aim at gaining further understanding of how emotional arousal relates to individual differences in behavioral and physiological reactivity and how the responses are interlinked. In particular, we question whether physiological responses during a novel object presentation and pre-feeding test are caused by behavioral changes, e.g., locomotion or, in the absence of behavioral activity or locomotion, by emotional arousal. Furthermore, we ask whether behavioral and physiological responses are stable across time and contexts. We expect that horses show a greater physiological reaction to a fear-inducing situation such as being exposed to a novel object, compared to the anticipatory pre-feeding experiment. Also, due to its greater salience, we expect horse physiological and behavioral responses to the novel object to be of greater similarity over time compared to the response of individuals to the pre-feeding excitement test, which would not represent an event that horses would often encounter in the wild.

## Materials and Methods

### Animals and Housing

The study was conducted at the equine yard of the College of West Anglia (United Kingdom) between July and November 2017. The research was conducted on 20 horses which were individually stabled in loose boxes. Five of the 20 horses were tested only in one of the two experimental conditions, two solely for pre-feeding excitement and three only for novel object test, due to their lack of availability during testing periods (Table 1). The sample included 14 geldings [age: mean 11.8 ± 3.8 years (yrs), range 6–18 yrs] and six mares (age: mean 11.8 ± 2.6 yrs, range 10–17 yrs) of diverse breeds, use, and training experiences (Table 1). The horses were fed twice a day: once in the morning (0800–0830) and once in the afternoon (1500–1600). Water was available *ad libitum*, and feces were removed from the stables after the horses were fed. In the late afternoon (1600–1700), some of the horses were turned out in the paddock for the night.

**Table 1:** Age in years, sex, and breed of the 20 horses tested for this study.

Age	Sex	Breed	NO1	NO2	PF1	PF2
6	Gelding	KWPN	36.67 ± 2.59	31.41 ± 1.52	42.10 ± 4.30	38.41 ± 2.57
6	Gelding	Cob	44.22 ± 9.89	39.63 ± 4.86	53.74 ± 8.18	37.48 ± 3.31
8	Gelding	Cob	44.54 ± 1.56	38.37 ± 0.40	41.08 ± 5.62	33.69 ± 2.48
9	Gelding	KWPN	43.87 ± 6.93	45.93 ± 12.30	39.01 ± 2.80	39.95 ± 7.07
9	Gelding	Irish Sports Pony	69.63 ± 15.71	62.12 ± 8.10	64.30 ± 12.74	36.77 ± 3.04
11	Gelding	Welsh Section A	62.73 ± 20.70	55.29 ± 25.91	*NA*	*NA*
12	Gelding	KWPN	54.30 ± 5.87	40.24 ± 8.05	*NA*	*NA*
13	Gelding	Cob	*NA*	*NA*	48.29 ± 9.89	57.14 ± 12.43
13	Gelding	Cob	*NA*	*NA*	55.57 ± 5.06	50.73 ± 1.53
14	Gelding	Welsh Pony	51.47 ± 8.87	51.21 ± 5.66	67.27 ± 12.41	54.42 ± 8.01
14	Gelding	Cob	46.82 ± 2.69	54.67 ± 3.85	51.29 ± 9.89	55.47 ± 10.65
16	Gelding	Shire X Warmblood	83.26 ± 20.00	80.03 ± 29.02	60.78 ± 7.80	46.88 ± 6.05
16	Gelding	Welsh Crossbred	71.47 ± 18.82	44.41 ± 2.40	68.63 ± 9.19	53.98 ± 13.29
18	Gelding	Thoroughbred	44.43 ± 8.47	36.94 ± 4.96	41.58 ± 1.78	33.59 ± 1.90
10	Mare	Cob	44.28 ± 12.98	66.93 ± 15.66	42.84 ± 4.98	44.89 ± 7.01
10	Mare	Welsh Crossbred	62.40 ± 12.40	43.16 ± 1.92	48.06 ± 5.75	65.76 ± 14.01
11	Mare	Warmblood	40.25 ± 4.11	42.52 ± 6.27	50.69 ± 17.53	38.71 ± 4.06
11	Mare	Appaloosa X Cob	42.84 ± 1.54	41.92 ± 8.30	60.89 ± 3.36	48.30 ± 12.39
12	Mare	Thoroughbred X Cob	43.93 ± 3.47	47.27 ± 4.77	61.14 ± 7.16	46.12 ± 3.40
17	Mare	Gypsy Cob	54.77 ± 9.71	45.16 ± 3.44	*NA*	*NA*

*Mean heart rate expressed in beats per minute (bpm) and standard deviation during the different experimental situations: novel object experiment (NO) and pre-feeding excitement test (PF). Numbers 1 and 2 indicate repetition of experiments, with number 1 indicated tests in summer (July/August) and number 2 tests in autumn (October/November). The color code indicates the horses being categorized as high (orange) or low (blue) behavioral responders.*

### Experimental Design

**FIGURE 1:**
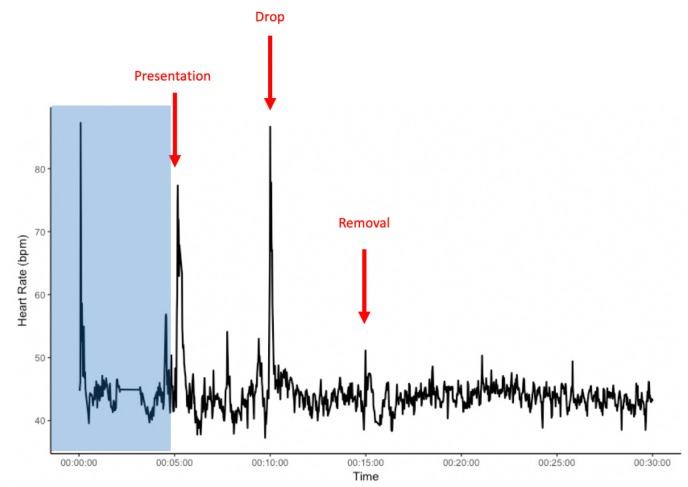
Example heart rate of an individual from the start of heart rate recordings and during the novel object experiment. The area shaded in blue presents a 5 min habituation period after the heart rate monitor is placed on the horse, but before the start of the novel object exposure. *x*-axis: time in minutes; *y*-axis: heart rate in beats per minute (bpm).

**FIGURE 2:**
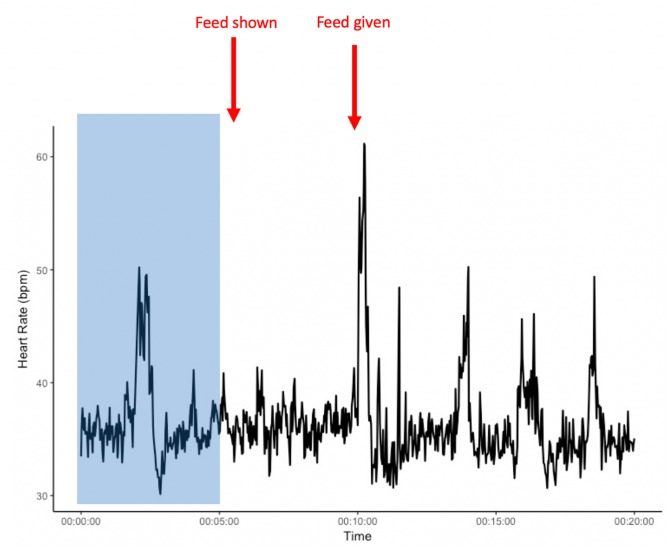
Example heart rate of an individual from the start of heart rate recordings and during the pre-feeding excitement experiment. The area shaded in blue presents a 5-min habituation period after the heart rate monitor is placed on the horse, but before the start of the experiment. *x*-axis: time in minutes; *y*-axis: heart rate in beats per minute (bpm).

We conducted two experimental tests – the pre-feeding excitement and the novel object test, presented in random order, with one trial per horse and repeated in summer (test 1 – July/August) and in autumn (test 2 – October/November). Behavior was recorded by video camera (Canon Legria HF R56), and HR was recorded using a Polar^®^ V800 system. The belt was placed around the chest of the horses, positioned where a saddle or vaulting girth would normally sit. The belt consists of an electrode belt with a built-in transmitter, connected via Bluetooth to a wristwatch (receiver). To optimize the contact between the belt and the skin, both the coat of the horse in the interested area and the belt were wetted. The receiver was placed at the stable entrance or inside the stable. All horses were already habituated to wearing the HR belt prior to the present study. Before each test, an adjustment period of 5 min was allowed to exclude potential effects of prior handling (Figure 1, 2).

In the novel object test, the horses were exposed to one of three different objects in their stable. The first object was formed of a main cylindrical hard body (approximately 30 cm in length and 7 cm in diameter) filled with gravel which was fixed to a soft foam rubber ball (about 15 cm in diameter) and covered in blue fabric. The second object was formed of two cylindrical plastic tubes fixed together to form an “x.” Similar to the first object, the cylinders were approximately 30 cm in length and 7 cm in diameter, filled with gravel and covered with yellow fabric. In addition, 12 tennis balls of different colors (green, blue, red, and yellow) and materials were pierced and attached to four strings (three balls per string) of approximately 50 cm in length. These were then tied to the main body of the object and left hanging. The third object was a pink inflatable guitar of approximately 1 m in length. All objects were attached to a string, around 4 m long, to allow their retrieval from the stable and are shown in Supplementary Figure S1.

**FIGURE 3:**
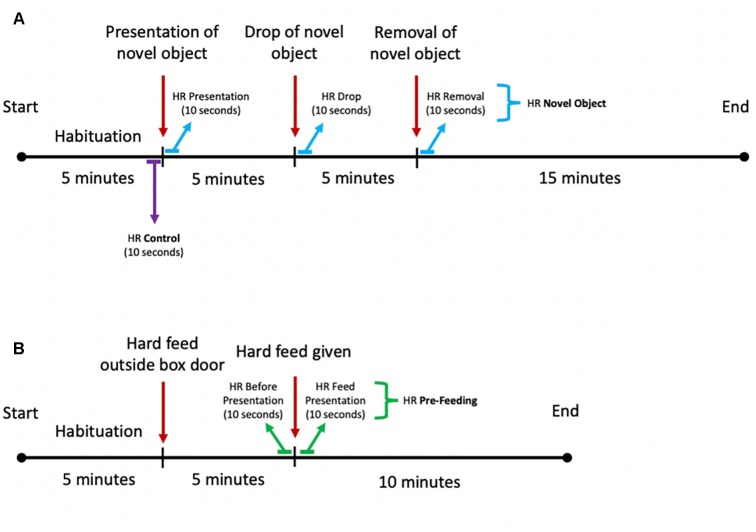
Schematic diagram of the experimental setup for the **(A)** novel object and **(B)** pre-feeding excitement paradigms.

The object assigned to each individual was randomized for each season as well as the order of the horses tested. For the second repeat, the object was chosen randomly from the remaining two objects. To avoid the horses seeing the object before testing, the objects were covered from sight when carried around the yard. The novel object tests took place between the hours of 0900 and 1300 and between 1500 and 1800 when the yard was quiet, and the horses were fed. The test was based on the procedure described by Górecka-Bruzda et al. (2011) and Dai et al. (2015) and adapted for the present experiment. A novel object was placed over the box entrance, with the cord hanging over the stable door to keep the object at the height of approximately 1 m. The object was kept in this position for the following 5 min and was then dropped to the floor (the objects filled with gravel created a muffled noise). The horse reaction was recorded for the following 5 min. Thereafter, the object was removed from the stable, while behavioral monitoring and HR measurement continued for another 15 min (Figure 3A).

The pre-feeding excitement test was conducted during morning feeds (0800–0830) and started with the horses being shown a bucket containing their individual mix of hard feed on the floor outside the stable, while the other horses were fed. The horses’ physiological and behavioral responses were recorded for 5 min. Thereafter, the horses were given their hard feed by placing the feeding bucket inside the box and their behavioral and physiological responses were measured for the following 10 min (Figure 3B).

### Data Processing

Raw HR data were purged with a moving average filter to remove biologically implausible outlier values. Due to the quick regulation of HR (von Borell et al., 2007), the following HR variables were calculated: (1) mean HR in beats per minute (bpm) for the 10 s preceding and following the introduction of the hard feed inside the box, as well as preceding and following the presentation, drop and removal of the object; (2) HR increase in bpm following the food introduction and novel object presentation, drop, and removal, calculated as difference between maximum value within 60 s from the exposure to the stimulus and 3 s average HR before the presentation of the stimulus. Such timeframes were selected in order to assess the immediate cardiac response of the horses to the stimulus, as well as to measure the degree of activation of the SAM, which can often appear only after 20–30 s after the detection of the stressor (von Borell et al., 2007). For each horse, we calculated three average HR values for the novel object experiments: one following the presentation of the object, one following the drop of the object, and one following the removal of the object. For the pre-feeding experiments, two average HR values were calculated: one before the hard feed was given to the horse and one after the introduction of the hard feed in the stable. For both novel object and pre-feeding experiments, control average HRs were calculated from the 10 s preceding the presentation of the novel object (Figure 3A).

Behavioral responses of the horses were analyzed from videos using Solomon Coder v. beta 17.03.22 (András Péter^1^) and an ethogram of the behaviors coded can be found in Supplementary Table S1. Behavior of the individuals was analyzed for the five minutes prior to the presentation of the hard feed for the pre-feeding excitement. For the novel object task, the 5 min following the presentation and drop of the object and the 2 min following its removal were analyzed. The behavior analyzed included walking, pawing, occurrence of vocalizations (snorting and whinnying), occurrence of startle response, and defecating as their frequency has been shown to increase in threatening and stress-inducing situations (Seaman et al., 2002; Lansade et al., 2008; Leiner and Fendt, 2011). Behavior was recorded as continuous variables, e.g., walking as duration of behavior in s per observation period, or frequency of behavior per observation period, e.g., snorting. The classification of the individual in high and low behavioral respondents was based on the frequency of vocalizations and duration of pawing behavior for the pre-feeding experiment; whereas on frequency of startle response, defecation, and vocalizations for the novel object experiment (Table 2). Specifically, horses were classified as high behavioral respondents in the pre-feeding experiment if they performed more than two vocalizations and/or more than 20 s of pawing. Horses that performed less than two vocalizations and/or less than 20 s of pawing during the pre-feeding experiment were categorized as low respondents. For the novel object test, horses were classified as high behavioral respondents if they performed a startle response and/or defecated and/or vocalized for more than four times. Horses were classified as low respondents in the novel object test if they performed less than four vocalizations, no startle response and no defecation. The vocalization threshold was increased for the subdivision of the horses in low and high respondent in the novel object test due to the exposure of the horses to more stimuli (presentation, drop, and removal of object) in this test, compared to the sole presentation of the hard feed in the pre-feeding test. Walking was recorded to assess effects of locomotion on HR responses.

**Table 2:** Description of behavioral categories (high vs low) and number of individuals per category, per test repeat.

Condition	Value	Description	*n*	*n*
			*(Test 1)*	*(Test 2)*
Pre-feeding	Low	Less than two vocalizations (snorting and/or whinnying) and less than 20 s of pawing behavior	8	11
	High	More than two vocalizations (snorting and/or whinnying) and/or pawing for more than 20 s	9	6
Novel object	Low	No defecation, no startle response, less than four vocalizations (snorting and/or whinnying)	9	14
	High	Defecation, and/or startle response, and/or more than four vocalizations (snorting and/or whinnying)	9	4

### Statistical Analysis

All data were analyzed using R version 3.4.3 (RStudio Team, 2016; R Core Team, 2017). In order to investigate how behavioral and physiological reactivity of horses varied across contexts and how such responses were interlinked, we conducted two generalized linear mixed models (GLMMs) with the additional packages “glmmADMB” (Skaug et al., 2016). The response variable was assigned to the 10-s average HR for the first model (GLMM1) and to the HR increase for the second (GLMM2). Both models had the same fixed factors, namely, the experimental situation (pre-feeding excitement, novel object or control), test number (first vs second), behavioral categorization (high versus low respondents), and locomotion (duration of walking), together with the interaction between experiment and behavioral response categorization, as well as the interaction between experiment and locomotion. For the purpose of the analysis, the different conditions of each experiment – such as the time before and after the presentation of the feed in the pre-feeding experiment, and the presentation, drop and removal of the object in the novel object experiment – were individually included in the dataset, resulting in horses having multiple values for each experiment. The “multicomp” (Hothorn et al., 2008) package was used to conduct the post hoc analysis. In particular, Tukey test for multiple comparisons was chosen to gain further understanding of the effect of the fixed factors in the models. We analyzed multicollinearity between fixed factors by calculating the variance inflation factors (VIFs) through the “vif” function in the package “car” (Fox and Weisberg, 2011). VIFs for both models were below 1.02, indicating no issue with multicollinearity being present (Zuur et al., 2009). A likelihood ratio test was used to compare models fit according to presence or absence of the individual random effect.

To analyze the consistency of both behavioral and physiological responses over time, we used the “rptR” package (Stoffel et al., 2017). In particular, we assessed the repeatability of the 10-s average HR and HR increase with 1000 permutations for the physiological reactivity data collected for the control, novel object, and pre-feeding conditions. The repeatability of behavioral categorization was assessed by coding with 1 the individuals showing a high behavioral response and 0 the horses performing little behavioral response and conducted with 1000 permutations for the novel object and pre-feeding experiments separately. The significance level was set at α = 0.05.

## Results

### Physiological and Behavioral Responses to Experimental Situations

#### Average Heart Rate

**FIGURE 4:**
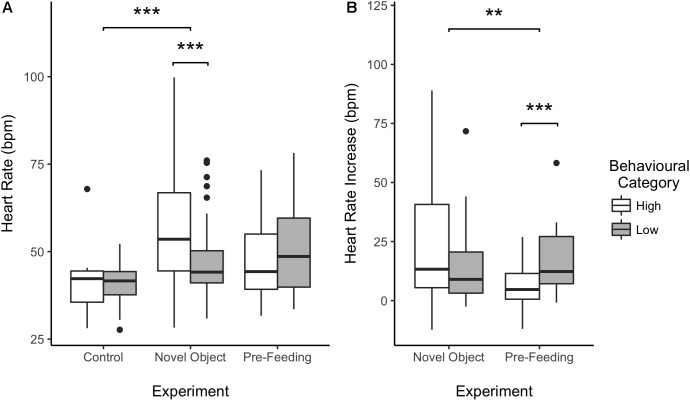
Effect of behavioral categorization on the mean heart rate (HR) **(A)** and HR increase **(B)** of the horses recorded during the study. High and low behavioral category indicates whether the individual a more or less intense behavioral response during the testing situation. Boxplots represent the median (black bar), the interquartile range – IQR (boxes), maximum and minimum values excluding outliers (whiskers) and outliers (black dots). ^∗∗^*p* < 0.01 and ^∗∗∗^*p* < 0.001.

**FIGURE 5:**
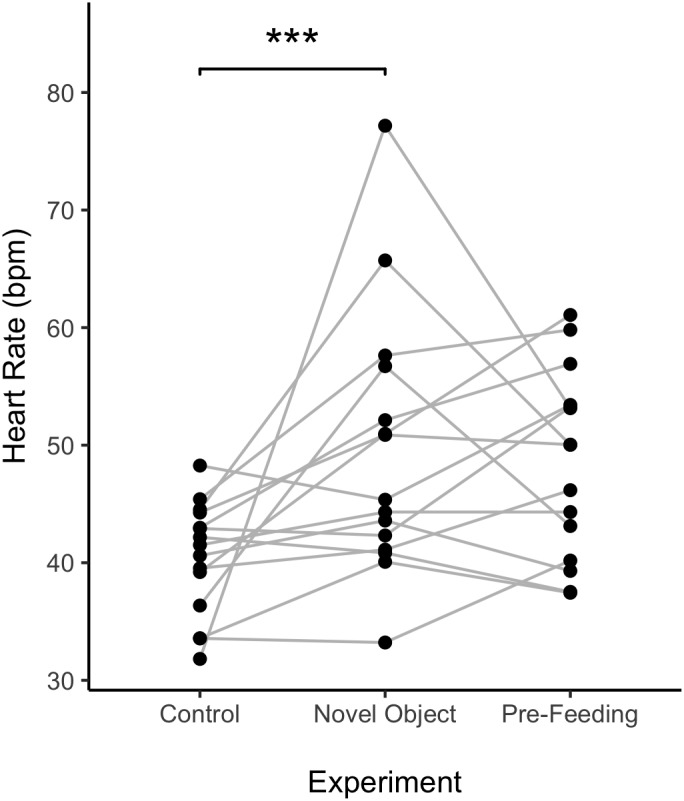
Individual average heart rate of horses across experiments. Points represent a value per individual horse and the lines connect individual horses over different experiments. ^∗∗∗^*p* < 0.001.

Average HR of the horses was significantly higher during the novel object experiment compared to the control period (Tukey: *z* = 4.980, *p* < 0.001; Figure 4A, 5) and tended to be higher during the pre-feeding excitement compared to the control period (Tukey: *z* = 2.104, *p* = 0.083; Figure 1A, 5). HR between novel object and pre-feeding excitement was not significantly different (Tukey: mean: *z* = −1.986, *p* = 0.108; Figure 4A, 5). We found a significant interaction between behavioral response categorization and experiment affecting HR. Average HR during the novel object experiment was significantly lower in the group of horses showing a low behavioral response compared to horses showing a high behavioral response (GLMM1: *z* = −3.66, *p* < 0.001; Figure 4A). Locomotion did not have any effect on the average HR of the horses (GLMM1: *z* = −0.77, *p* = 0.439) and the average HR model including individual identity as random factor had a significantly better fit compared to the model without the random effect (ANOVA: deviance = 5.688, df = 1, *p* = 0.017). Full model results can be found in Table 3.

**Table 3:** Results of the first full generalized linear mixed model investigating the factors affecting patterns in average HR of horses.

Parameters	Estimate ± SE	*z*	*p*
**Intercept**	42.885 ± 3.139	13.65	<0.001
**Experiment (*novel object*)**	18.046 ± 3.623	4.98	<0.001
**Experiment (*pre-feeding*)**	7.686 ± 3.653	2.10	0.035
Test repeat	−2.863 ± 1.539	−1.86	0.063
Walking	−3.019 ± 3.902	−0.77	0.439
Behavioral category	−0.453 ± 3.825	−0.12	0.906
Experiment (*novel object*) ^∗^ walking	3.726 ± 3.949	0.94	0.345
Experiment (*pre-feeding*) ^∗^ walking	5.797 ± 4.266	1.36	0.174
**Experiment (*novel object*) ^∗^ behavioral category**	−15.601 ± 4.260	−3.66	<0.001
Experiment (*pre-feeding*) ^∗^ behavioral category	−1.203 ± 4.655	−0.26	0.796

*Factors with significant effects (*p* < 0.05) are highlighted in bold.*

#### Heart Rate Increase

Despite there being no difference between average HRs in the two experimental conditions, horses showed a significantly lower HR increase in the pre-feeding experiment (GLMM2: *z* = −2.97, *p* = 0.003; Figure 4B). Contrarily to what was seen in the average HR data, horses showing a low behavioral response were observed of having a higher HR increase compared to individuals with a high behavioral response (GLMM2: *z* = 3.34, *p* < 0.001; Figure 4B). Locomotion affected the HR increase of the subjects (GLMM2: *z* = 2.61, *p* = 0.009). Nonetheless, its interaction with experiment did not affect the data (GLMM2: *z* = −1.36, *p* = 0.174). Finally, the fit of the HR increase model having subject identity as a random factor did not vary from that of the model without the random effect (ANOVA: deviance = 1.05, df = 1, *p* = 0.306). Full model results can be found in Table 4.

**Table 4:** Results of the second full generalized linear mixed model investigating the factors affecting patterns in HR increase of horses.

Parameters	Estimate ± SE	*Z*	*p*
**Intercept**	23.603 ± 3.631	6.50	<0.001
**Experiment**	−16.278 ± 5.479	−2.97	0.003
Test repeat	4.563 ± 3.020	1.51	0.131
Walking	2.565 ± 0.983	2.61	0.009
**Behavioral category**	−18.277 ± 4.056	−4.51	<0.001
Experiment ^∗^ walking	−5.173 ± 3.801	−1.36	0.174
**Experiment ^∗^ behavioral category**	23.793 ± 7.126	3.34	<0.001

*Factors with significant effects (p < 0.05) are highlighted in bold.*

### Overall and Individual Repeatability of Physiological and Behavioral Responses

As a group, horses showed an overall tendency for mean HR to be higher during the first experimental session compared to the second repeat (GLMM1: *z* = −1.86, *p* = 0.063). Conversely, at the individual level, horses’ average HR was significantly repeatable across both experiments, showing how individual horses were consistent in their average HR response across repeats. In particular, individual horses’ average HR was more consistent during the novel object experiment (*R* = 0.372, CI 95% [0.129, 0.575], *p* = 0.001), compared to the average HR of horses during the pre-feeding test (*R* = 0.221, CI 95% [0, 0.467], *p* = 0.022).

Similarly, HR increase was not significantly different between the two experimental repeats (GLMM2: *z* = 1.51, *p* = 0.131). Nonetheless, at the individual level, only the HR increase during the novel object test was significantly repeatable (*R* = 0.386, CI 95% [0.142, 0.572], *p* = 0.001), with the HR increase of individual horses during the pre-feeding experiment not being repeatable, and therefore not consistent, across test repeats (*R* = 0, CI 95% [0, 0.443], *p* = 1).

Out of the 18 horses tested in the novel object experiment, 11 showed consistent behavioral response between the two repeats. Of these 11, eight consistently showed a low behavioral response and three a high behavioral response. For the pre-feeding experiment, 12 horses showed a constant response across repeats, five of which were categorized as high behavioral respondent and seven as low behavioral respondent. Overall, nine horses performed a constant behavioral response in both experiments, of which only four horses being consistent in their behavioral response across experiments and repeats, and five showing opposite responses in the two experimental tests (Table 1). Nonetheless, the analysis showed how behavioral categorization of horses was not significantly repeatable across both novel object test and pre-feeding test (NO: *R* = 0.185, CI 95% [0, 0.586], *p* = 0.331; PF: *R* = 0.194, CI 95% [0, 0.429], *p* = 0.289).

## Discussion

In the present study, we investigated individual behavioral and physiological responses of horses during two experimental procedures, a novel object experiment (NO) and a pre-feeding test (PF). We found a higher HR increase in response to the NO test compared to the PF test. Furthermore, our results suggest that in a fearful context (NO) behavioral arousal was linked to a higher physiological arousal, whereas in a feeding context, the relationship between behavioral and physiological arousal was less pronounced. This is in line with previous studies describing that individuals classified as calmer had higher HR compared to more behaviorally excited individuals when tested for pre-feeding reactivity (horses: Jezierski et al., 1999; Christensen et al., 2005; Lansade et al., 2008; Ellis et al., 2014; cattle: Welp et al., 2004).

Based on our analysis, we found rather little behavioral consistency between test repeats and only a limited number of individuals responded similarly across contexts and repeats, which would have been expected when behavioral responses in experimental tests are indicative of temperamental traits. Conversely, average HR was significantly repeatable across both experiments. In particular, in line with our predictions, the horses’ physiological responses were more repeatable for the NO experiment compared to the PF test. Similarly, the HR increase resulting from the NO experiment was consistent across repeats, while it was not repeatable for the PF test. Such variation reflected how behavioral responses of horses do not necessarily predict physiological reactions during a NO and PF excitement test. This marked distinction in repeatability of behavioral and physiological responses highlight how current methods of behavioral classification of horse temperament may not be as appropriate as previously thought. In fact, despite the two experiments measuring the horse responses in two different contexts, we would have expected their behavioral responses to be stable at least across time if not across contexts, as the behaviors selected in our research are often used in the assessment of horse personality (Seaman et al., 2002; Lansade et al., 2008; Leiner and Fendt, 2011). Moreover, the disjointed results of the lack of repeatability of the behavior and general consistency of both HR indices of the horses across time suggest that behavior and physiological response may be decoupled and regulated independently. Classical models regarding individual differences in behavior and physiology assume them to be associated with each other to form different coping styles (Koolhaas et al., 1999). However, evidence for independent modulation of the HPA axis (Ferrari et al., 2013; Boulton et al., 2015; Dosmann et al., 2015), the SAM axis (Qu et al., 2018), and behavioral traits have been recently accumulating. For example, Harewood and McGowan (2005) showed how stabling naïve horses resulted in an elevated performance of stress-related behaviors, which did not correlate with changes in HR or salivary cortisol, contradicting previous literature (Goldsmith et al., 1987; Le Scolan et al., 1997; Momozawa et al., 2005; Lansade et al., 2008; Grajfoner et al., 2010).

In our NO experiment, we were able to exclude a possible effect of locomotion on the HR of the subjects, allowing us to link the physiological response to underlying emotional arousal. Conversely, during the PF task individuals lacking a strong behavioral response during the experiment showed a higher HR increase, which indicates that emotional arousal, but not physical activity, accounted for the increase. Effects of emotional arousal on physiological responses have already been identified in other non-human animals. For example, in the study by Wascher et al. (2008), immobile greylag geese (*Anser anser*) watching aggressive interaction between conspecifics showed a significantly higher increase in HR compared to geese watching non-social interactions. Moreover, an increase in physiological reactivity resulting solely from emotional arousal was also identified in guide dogs (Fallani et al., 2007). Conversely, horses that were classified as high behavioral responders during the PF experiments had and once they were given the feed, despite having similar average HRs to low behaviorally respondent horses. Identifying emotional valence from arousal in different contexts has proved to be challenging, especially in experiments aiming at detecting positive emotional states (Reefmann et al., 2009). High emotional arousal triggers mechanisms of increased attention and energy mobilization to prepare the subject to cope with an adverse situation, facilitating a possible fight-or-flight response (Dawkins, 1998). Conversely, positive states, such as feeding or grazing, tend to show a physiologically lower arousal levels compared to negative states, with some exceptions, e.g., sexual activity. Reefmann et al. (2009) suggest how in sheep, behavioral responses together with physiological ones may aid to identify the valence of emotional states in animals. Therefore, both behavioral and physiological responses are needed for a conclusive assessment of individual emotional reactivity, as we have shown that the relationship between the two is not stable over time or contexts.

In some situations, showing emotional arousal behaviorally may represent an important adaptation in group-living species. While some behaviors may simply result from sympathetic activation, such as defecation (Van Reenen et al., 2005), others can signal important information, e.g., danger, to group members (Špinka, 2012; Maigrot et al., 2017). The social aspect of emotion has been extensively studied in humans (Bastiaansen et al., 2009; Špinka, 2012), with studies showing how arousal can promote information sharing (Berger, 2011). In animals, however, research is still lacking. Pigs (*Sus scrofa*) have been focus of attention regarding emotionally driven behavior, with studies providing information on how specific pitches of vocalizations not only were related to heightened arousal but were also specific to the negative valence of the emotion felt (Düpjan et al., 2008). Similar findings were shown in horses, with Maigrot et al. (2017) providing further information regarding vocalizations deriving from emotional arousal in both Przewalski’s (*Equus przewalskii*) and domestic horses. Such findings highlight how emotionally driven behaviors can represent reliable indicators of precise emotional/motivational states which can play a key role in group-living species in avoiding danger. As group-living species rely on group coordination to survive, providing information to others about an individual’s emotional arousal can allow for a better coordination in avoiding negative aspects of an environment (Spoor and Kelly, 2004). In fact, it is thought that negative emotions related to high arousal, such as fear, ought to spread more quickly than positive ones due to the urgent nature of the signal (Špinka, 2012). The results from our study support such hypothesis. We have showed how the performance of behaviors linked to high arousal varies according to context. In the NO condition, horse behavioral response was linked with their physiological arousal, whereas in the PF experiment, individual emotional arousal did not match an increased performance of stress-related behaviors. Such difference may arise from the perceptually different stimuli, providing evidence that sharing arousal-related information is more likely to happen in a fearful situation to aid conspecific coordination. On the contrary, the conditions and stimuli during the PF experiment may not represent evolutionarily salient stressors, reducing the need of expressing emotional arousal behaviorally. In fact, in the wild horses are less likely to suffer from food shortages compared to the risk of being attacked by a potential predator.

Overall, the results of the present research must be handled cautiously due to the low sample size: only 15 out of 20 horses were tested in both testing conditions (NO and PF) and periods. Furthermore, we did not test for potential effects of sex, age, or breed. To conclude, our study suggests independencies between physiological and behavioral reactions in non-fearful contexts as well as a low repeatability of behavioral, contrarily to physiological, responses in different test conditions over time. To gain a more conclusive insight into individual differences regarding behavioral and physiological response patterns, we would suggest to combine tests across different experimental contexts. Especially our findings from the PF test indicate that models, such as coping style, which were derived from studies in the context of fear and aggression, might not translate to other contexts.

## Ethics Statement

All applied methods were non-invasive, and the experimental procedure was approved by Anglia Ruskin University’s Departmental Research Ethics Panel (Reference Number: A&EB DREP 17-053).

## Author Contributions

AS, DH, and CW designed the experiments and wrote the manuscript. AS and DH conducted the experiments. AS and CW analyzed the data.

## Conflict of Interest Statement

The authors declare that the research was conducted in the absence of any commercial or financial relationships that could be construed as a potential conflict of interest.

## Footnotes

1 www.solomoncoder.com

## References

[B1] Albuquerque N. Guo K. Wilkinson A. Resende B. Mills D. (2018). Mouth-licking by dogs as a response to emotional stimuli. *Behav. Process.* 146 42–45. 10.1016/j.beproc.2017.11.006 29129727

[B2] Anderson C. Yngvesson J. Boissy A. Uvnäs-Moberg K. Lidfors L. (2015). Behavioural expression of positive anticipation for food or opportunity to play in lambs. *Behav. Process.* 113 152–158. 10.1016/j.beproc.2015.02.003 25659525

[B3] Anderson D. J. Adolphs R. (2014). A framework for studying emotions across species. *Cell* 157 187–200. 10.1016/j.cell.2014.03.003 24679535 PMC4098837

[B4] Barnard S. Matthews L. Messori S. Podaliri-Vulpiani M. Ferri N. (2015). Laterality as an indicator of emotional stress in ewes and lambs during a separation test. *Anim. Cogn.* 19 207–214. 10.1007/s10071-015-0928-3 26433604

[B5] Bastiaansen J. Thioux M. Keysers C. (2009). Evidence for mirror systems in emotions. *Philoso. Trans. R. Soc. B Biol. Sci.* 364 2391–2404. 10.1098/rstb.2009.0058 19620110 PMC2865077

[B6] Bennett V. Gourkow N. Mills D. (2017). Facial correlates of emotional behaviour in the domestic cat (*Felis catus*). *Behav. Process.* 141 342–350. 10.1016/j.beproc.2017.03.01 28341145

[B7] Berger J. (2011). Arousal increases social transmission of information. *Psychol. Sci.* 22 891–893. 10.1177/0956797611413294 21690315

[B8] Boulton K. Couto E. Grimmer A. J. Earley R. L. Canario A. V. M. Wilson A. J. (2015). How integrated are behavioral and endocrine stress response traits? A repeated measures approach to testing the stress-coping style model. *Ecol. Evol.* 5 618–633. 10.1002/ece3.1395 25691986 PMC4328767

[B9] Briefer E. F. Tettamanti F. McElligott A. G. (2015). Emotions in goats: mapping physiological, behavioural and vocal profiles. *Anim. Behav.* 99 131–143. 10.1016/j.anbehav.2014.11.002

[B10] Bulmer L. McBride S. Williams K. Murray J. (2015). The effects of a high-starch or high-fibre diet on equine reactivity and handling behaviour. *Appl. Anim. Behav. Sci.* 165 95–102. 10.1016/j.applanim.2015.01.008

[B11] Carere C. Groothuis T. G. Möstl E. Daan S. Koolhaas J. M. (2003). Fecal corticosteroids in a territorial bird selected for different personalities: daily rhythm and the response to social stress. *Horm. Behav.* 43 540–548. 10.1016/S0018-506X(03)00065-5 12799170

[B12] Carere C. van Oers K. (2004). Shy and bold great tits (*Parus major*): body temperature and breath rate in response to handling stress. *Psychol. Behav.* 82 905–912. 10.1016/j.physbeh.2004.07.009 15451657

[B13] Carter A. J. Feeney W. E. Marshall H. H. Cowlishaw G. Heinsohn R. (2013). Animal personality: what are behavioural ecologists measuring? *Biol. Rev.* 88 465–475. 10.1111/brv.12007 23253069

[B14] Christensen J. W. Keeling L. J. Nielsen B. L. (2005). Responses of horses to novel visual, olfactory and auditory stimuli. *Appl. Anim. Behav. Sci.* 93 53–65. 10.1016/j.applanim.2005.06.017

[B15] Chrousus G. P. Gold P. W. (1992). The concepts of stress and stress system disorders. Overview of physical and behavioral homeostasis. *J. Am. Med. Assoc.* 267 1244–1252. 10.1001/jama.1992.03480090092034 1538563

[B16] Claessens S. E. Daskalakis N. P. van der Veen R. Oitzl M. S. de Kloet E. R. Champagne D. L. (2011). Development of individual differences in stress responsiveness: an overview of factors mediating the outcome of early life experiences. *Psychopharmacology* 214 141–154. 10.1007/s00213-010-2118-y 21165737 PMC3045508

[B17] Cockrem J. F. (2007). Stress, corticosterone responses and avian personalities. *J. Ornithol.* 148 169–178. 10.1007/s10336-007-0175-8

[B18] Dai F. Cogi N. H. Heinzl E. U. L. Dalla Costa E. Canali E. Minero M. (2015). Validation of a fear test in sport horses using infrared thermography. *J. Vet. Behav. Clin. Appl. Res.* 10 128–136. 10.1016/j.jveb.2014.12.001

[B19] Dawkins M. (1998). Evolution and animal welfare. *Quart. Rev. Biol.* 73 305–328. 10.1086/420307 9737005

[B20] Dosmann A. J. Brooks K. C. Mateo J. M. (2015). Within-individual correlations reveal link between a behavioral syndrome, condition and cortisol in free-ranging Belding’s ground squirrels. *Ethology* 121 125–134. 10.1111/eth.12320 25598565 PMC4295653

[B21] Drent P. J. van Oers K. van Noordwijk A. J. (2003). Realized heritability of personalities in the great tit (*Parus major*). *Proc. R. Soc. Lond. Ser. B Biol. Sci.* 270 45–51. 10.1098/rspb.2002.2168 12590770 PMC1691215

[B22] Düpjan S. Schön P. Puppe B. Tuchscherer A. Manteuffel G. (2008). Differential vocal responses to physical and mental stressors in domestic pigs (*Sus scrofa*). *Appl. Anim. Behav. Sci.* 114 105–115. 10.1016/j.applanim.2007.12.005

[B23] Ellis A. D. Stephenson M. Preece M. Harris P. (2014). A novel approach to systematically compare behavioural patterns between and within groups of horses. *Appl. Anim. Behav. Sci.* 161 60–74. 10.1016/j.applanim.2014.09.017

[B24] Fallani G. Previde E. P. Valsecchi P. (2007). Behavioral and physiological responses of guide dogs to a situation of emotional distress. *Physiol. Behav.* 90 648–655. 10.1016/j.physbeh.2006.12.001 17234220

[B25] Ferrari C. Pasquaretta C. Carere C. Cavallone E. von Hardenberg A. Réale D. (2013). Testing for the presence of coping styles in a wild mammal. *Anim. Behav.* 85 1385–1396. 10.1016/j.anbehav.2013.03.030

[B26] Finlayson K. Lampe J. Hintze S. Würbel H. Melotti L. (2016). Facial indicators of positive emotions in rats. *PLoS One* 11:e0166446. 10.1371/journal.pone.0166446 27902721 PMC5130214

[B27] Flaherty C. Rowan G. (1989). Rats (*Rattus norvegicus*) selectively bred to differ in avoidance behavior also differ in response to novelty stress, in glycemic conditioning, and in reward contrast. *Behav. Neural Biol.* 51 145–169. 10.1016/S0163-1047(89)90782-6 2649069

[B28] Fox J. Weisberg S. (2011). *An {R} Companion to Applied Regression*, 2nd Edn. California, CA: Sage Publications.

[B29] Fureix C. Pagès M. Bon R. Lassalle J. M. Kuntz P. Gonzalez G. (2009). A preliminary study of the effects of handling type on horses’ emotional reactivity and the human-horse relationship. *Behav. Process.* 82 202–210. 10.1016/j.beproc.2009.06.012 19591910

[B30] Goldsmith H. Buss A. Plomin R. Rothbart M. Thomas A. Chess S. (1987). Roundtable: what is temperament? Four approaches. *Child Dev.* 58 505–529. 10.2307/1130527 3829791

[B31] Górecka-Bruzda A. Jastrzêbska E. Sosnowska Z. Jaworski Z. Jezierski T. Chruszczewski M. H. (2011). Reactivity to humans and fearfulness tests: field validation in Polish cold blood horses. *Appl. Anim. Behav. Sci.* 133 207–215. 10.1016/j.applanim.2011.05.011

[B32] Grajfoner D. D. Austin E. J. Wemelsfelder F. (2010). Horse personality profiles and performance. *J. Vet. Behav. Clin. Appl. Res.* 5 26–27. 10.1016/j.jveb.2009.10.035

[B33] Harewood E. McGowan C. (2005). Behavioral and physiological responses to stabling in naive horses. *J. Equine Vet. Sci.* 25 164–170. 10.1016/j.jevs.2005.03.008

[B34] Hausberger M. Bruderer C. Le Scolan N. Pierre J. S. (2004). Interplay between environmental and genetic factors in temperament/personality traits in horses (*Equus caballus*). *J. Comp. Psychol.* 118 434–446. 10.1037/0735-7036.118.4.434 15584780

[B35] Hothorn T. Bretz F. Westfall P. (2008). Simultaneous inference in general parametric models. *Biomet. J.* 50 346–363. 10.1002/bimj.200810425 18481363

[B36] Houpt K. A. Hintz H. (1983). Some effects of maternal deprivation on maintenance behavior, spatial relationships and responses to environmental novelty in foals. *Appl. Anim. Ethol.* 76 297–307. 10.1016/0304-3762(83)90002-0

[B37] Jezierski T. Jaworski Z. Górecka- Bruzda A. (1999). Effects of handling on behaviour and heart rate in Konik horses: comparison of stable and forest reared youngstock. *Appl. Anim. Behav. Sci.* 62 1–11. 10.1016/S0168-1591(98)00209-3

[B38] König von Brostel U. Euent S. Graf P. König S. Gauly M. (2011). Equine behaviour and heart rate in temperament tests with or without rider or handler. *Physiol. Behav.* 104 454–463. 10.1016/j.physbeh.2011.05.010 21616087

[B39] Koolhaas J. M. Korte S. M. De Boer S. F. Van Der Vegt B. J. Van Reenen C. G. Hopster H. (1999). Coping styles in animals: current status in behavior and stress- physiology. *Neurosci. Biobehav. Rev.* 23 925–935. 10.1016/S0149-7634(99)00026-3 10580307

[B40] Lansade L. Bouissou M. Erhard H. W. (2008). Fearfulness in horses: a temperament trait stable across time and situations. *Appl. Anim. Behav. Sci.* 115 182–200. 10.1016/j.applanim.2008.06.011

[B41] Le Scolan N. Hausberger M. Wolff A. (1997). Stability over situations in temperamental traits of horses as revealed by experimental and scoring approaches. *Behav. Process.* 41 257–266. 10.1016/S0376-6357(97)00052-1 24896858

[B42] Leiner L. Fendt M. (2011). Behavioural fear and heart rate responses of horses after exposure to novel objects: effects of habituation. *Appl. Anim. Behav. Sci.* 131 104–109. 10.1016/j.applanim.2011.02.004

[B43] Lloyd A. S. Martin J. E. Bornett-Gauci H. L. Wilkinson R. G. (2008). Horse personality: variation between breeds. *Appl. Anim. Behav. Sci.* 112 369–383. 10.1016/j.applanim.2007.08.010

[B44] Maigrot A. Hillmann E. Anne C. Briefer E. (2017). Vocal expression of emotional valence in Przewalski’s horses (*Equus przewalskii*). *Sci. Rep.* 7:11. 10.1038/s41598-017-09437-1 28821880 PMC5562828

[B45] McCall C. A. Hall S. McElhenney W. H. Cummins K. A. (2006). Evaluation and comparison of four methods of ranking horses based on reactivity. *Appl. Anim. Behav. Sci.* 96 115–127. 10.1016/j.applanim.2005.04.021

[B46] Meaney M. J. (2001). Maternal care, gene expression, and the transmission of individual differences in stress reactivity across generations. *Annu. Rev. Neurosci.* 24 1161–1192. 10.1146/annurev.neuro.24.1.1161 11520931

[B47] Moberg G. P. (1985). “Biological response to stress: key to assessment of animal well-being?,” in *Animal Stress*, ed. Moberg G. P. (New York, NY: Springer).

[B48] Momozawa Y. Kusunose R. Kikusui T. Takeuchi Y. Mori Y. (2005). Assessment of equine temperament questionnaire by comparing factor structure between two separate surveys. *Appl. Anim. Behav. Sci.* 92 77–84. 10.1016/j.applanim.2004.11.006

[B49] Momozawa Y. Ono K. Sato F. Kikusui T. Takeuchi Y. Mori Y. (2003). Assessment of equine temperament by a questionnaire survey to caretakers and evaluation of its reliability by simultaneous behavior test. *Appl. Anim. Behav. Sci.* 84 127–138. 10.1016/j.applanim.2003.08.001

[B50] Olczak K. Winther Christensen J. Klocek C. (2018). Food motivation in horses appears stable across different test situations. *Appl. Anim. Behav. Sci.* 204 60–65. 10.1016/j.applanim.2018.04.006

[B51] Qu J. Fletcher Q. E. Réale D. Li W. Zhang Y. (2018). Independence between coping style and stress reactivity in plateau pika. *Physiol. Behav.* 197 1–8. 10.1016/j.physbeh.2018.09.007 30236525

[B52] R Core Team (2017)). *R: A Language and Environment for Statistical Computing.* Vienna: R Foundation for Statistical Computing.

[B53] Reefmann N. Wechsler B. Gygax L. (2009). Behavioural and physiological assessment of positive and negative emotion in sheep. *Anim. Behav.* 78 651–659. 10.1016/j.anbehav.2009.06.015

[B54] RStudio Team (2016)). *RStudio: Integrated Development for R.* Boston, MA: RStudio, Inc.

[B55] Russell J. A. (1980). A circumplex model of affect. *J. Pers. Soc. Psychol.* 39 1161–1178. 10.1037/h0077714

[B56] Schmidt A. Biau S. Möstl E. Becker-Birck M. Morillon B. Aurich J. (2010a). Changes in cortisol release and heart rate variability in sport horses during long-distance road transport. *Domest. Anim. Endocrinol.* 38 179–189. 10.1016/j.domaniend.2009.10.002 19962266

[B57] Schmidt A. Möstl E. Wehnert C. Aurich J. Müller J. Aurich C. (2010b). Cortisol release and heart rate variability in horses during road transport. *Hormo. Behav.* 57 209–215. 10.1016/j.yhbeh.2009.11.003 19944105

[B58] Seaman S. Davidson H. Waran N. (2002). How reliable is temperament assessment in the domestic horse (*Equus caballus*)? *Appl. Anim. Behav. Sci.* 78 175–191. 10.1016/s0168-1591(02)00095-3

[B59] Skaug H. Fournier D. Bolker B. Magnusson A. Nielsen A. (2016). *Generalized Linear Mixed Models Using ‘AD Model Builder’_. R Package Version 0.8.3.3.* Available at: https://rdrr.io/rforge/glmmADMB/ [accessed May 31, 2017].

[B60] Špinka M. (2012). Social dimension of emotions and its implication for animal welfare. *Appl. Anim. Behav. Sci.* 138 170–181. 10.1016/j.applanim.2012.02.005

[B61] Spoor J. R. Kelly J. R. (2004). The evolutionary significance of affect in groups: communication and group bonding. *Group Processes Intergroup Relat.* 7 398–412. 10.1177/1368430204046145

[B62] Stamps J. A. (2003). Behavioural processes affecting development: tinbergen’s fourth question comes of age. *Anim. Behav.* 66 1–13. 10.1006/anbe.2003.2180

[B63] Stoffel M. A. Nakagawa S. Schielzeth H. (2017). rptR: repeatability estimation and variance decomposition by generalized linear mixed-effects models. *Methods Ecol. Evol.* 8 1639–1644. 10.1111/2041-210X.12797

[B64] Van Reenen C. O’Connell N. Van der Werf J. Korte S. Hopster H. Jones R. (2005). Responses of calves to acute stress: individual consistency and relations between behavioral and physiological measures. *Physiol. Behav.* 85 557–570. 10.1016/j.physbeh.2005.06.015 16081113

[B65] Visser E. K. Ellis A. D. van Reenen C. G. (2008). The effect of two different housing conditions on the welfare of young horses stabled for the first time. *Appl. Anim. Behav. Sci.* 114 521–533. 10.1016/j.applanim.2008.03.003

[B66] Visser E. K. van Reenen C. G. Hopster H. Schilder M. B. H. Knaap J. Barneveld A. (2001). Quantifying aspects of young horses’ temperament: consistency of behavioural variables. *Appl. Anim. Behav. Sci.* 74 241–258. 10.1016/S0168-1591(01)00177-0

[B67] Visser E. K. van Reenen C. G. Schilder M. B. H. Barneveld A. Blokhuis H. J. (2003). Learning performances in young horses using two different learning tests. *Appl. Anim. Behav. Sci.* 80 311–326. 10.1016/S0168-1591(02)00235-6

[B68] Visser E. K. van Reenen C. G. van der Werf J. Schilder M. B. H. Knaap J. Barneveld A. (2002). Heart rate and heart rate variability during a novel object test and a handling test in young horses. *Physiol. Behav.* 76 289–296. 10.1016/S0031-9384(02)00698-4 12044602

[B69] von Borell E. Langbein J. Després G. Hansen S. Leterrier C. Marchant-Forde J. (2007). Heart rate variability as a measure of autonomic regulation of cardiac activity for assessing stress and welfare in farm animals: a review. *Physiol. Behav.* 92 293–316. 10.1016/j.physbeh.2007.01.007 17320122

[B70] Wascher C. A. F. Scheiber I. B. R. Kotrschal K. (2008). Heart rate modulation in bystanding geese watching social and non-social events. *Proc. R. Soc. B Biol. Sci.* 275 1653–1659. 10.1098/rspb.2008.0146 18430645 PMC2602813

[B71] Welp T. Rushen J. Kramer D. L. Festa-Bianchet M. de Passille A. M. (2004). Vigilance as a measure of fear in dairy cattle. *Appl. Anim. Behav. Sci.* 87 1–13. 10.1016/j.applanim.2003.12.013

[B72] Zuur A. F. Ieno E. N. Walker N. J. Saveliev A. A. Smith G. M. (2009). *Mixed Effects Models and Extension in Ecology With R.* New York, NY: Springer.

