# Supplementary material for: Testing for Behavioral and Physiological Responses of Domestic Horses (Equus caballus) Across Different Contexts – Consistency Over Time and Effects of Context
Source: Front Psychol. 2019 Apr 18;10:849. doi: 10.3389/fpsyg.2019.00849 (PMC6482254; doi:10.3389/fpsyg.2019.00849)
Supplement: Supplementary file 1 [file Table_1.docx]

Testing for behavioural and physiological responses of domestic horses (*Equus caballus*) across different contexts - consistency over time and effects of context

# Alexandra Safryghin, Denise V. Hebesberger & Claudia A.F. Wascher*


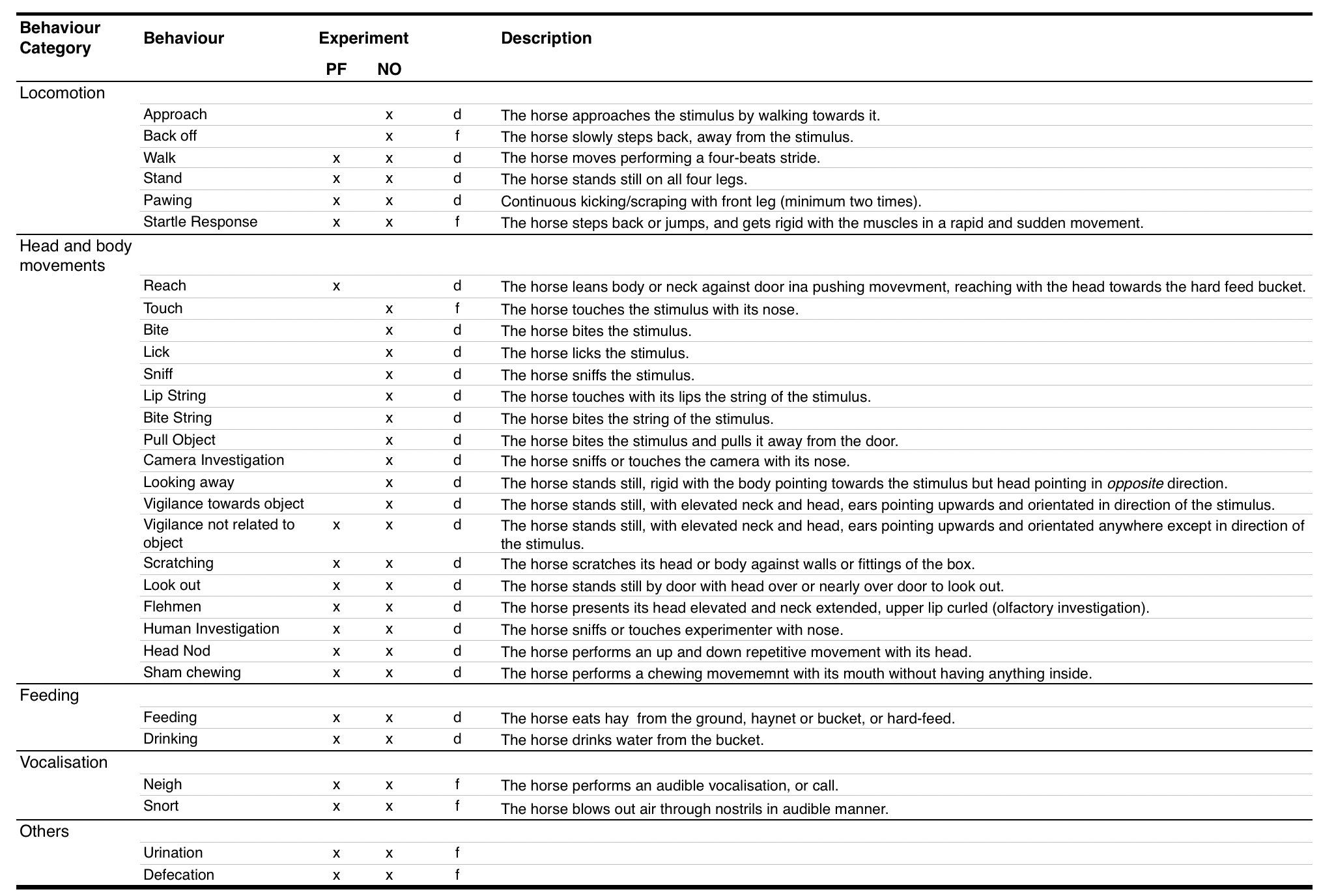
Supplementary Table 1: Ethogram of behaviours coded. Crosses represent whether the behaviour was coded for the pre-feeding experiment (PF) and/or the novel-object experiment (NO). It is also specified whether the behaviour was recorded as frequency (f) or as duration (d).


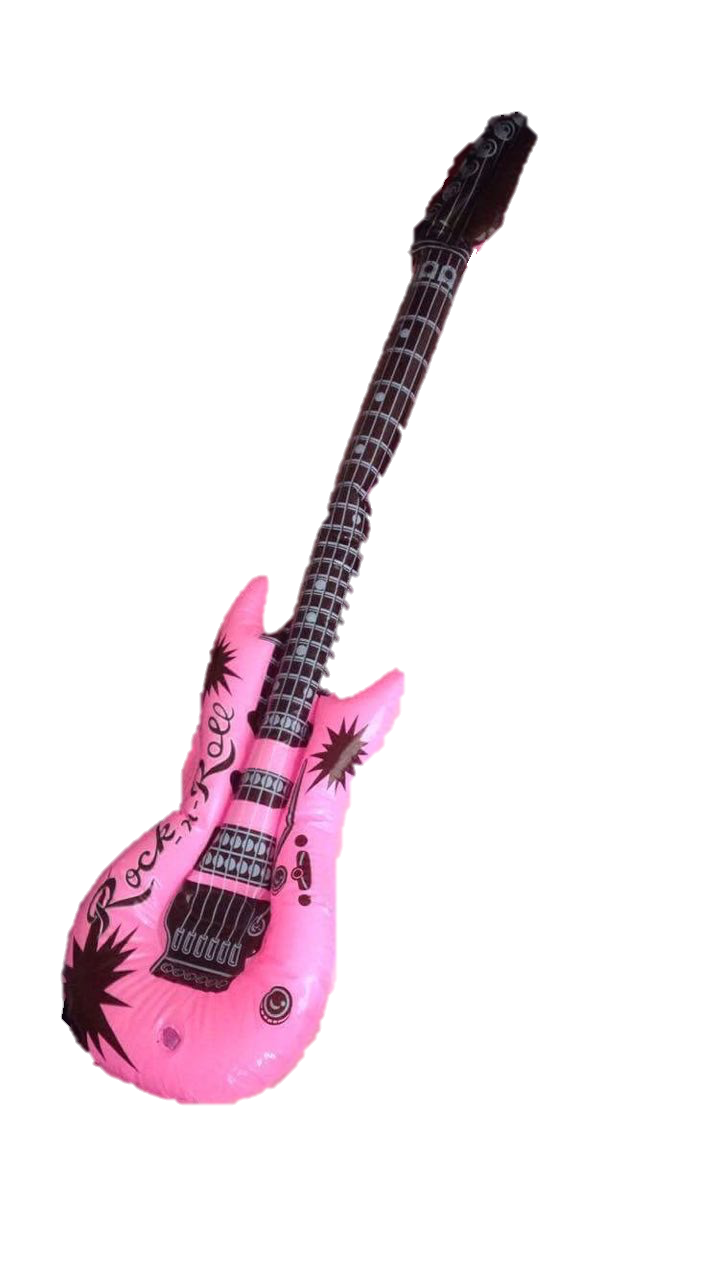

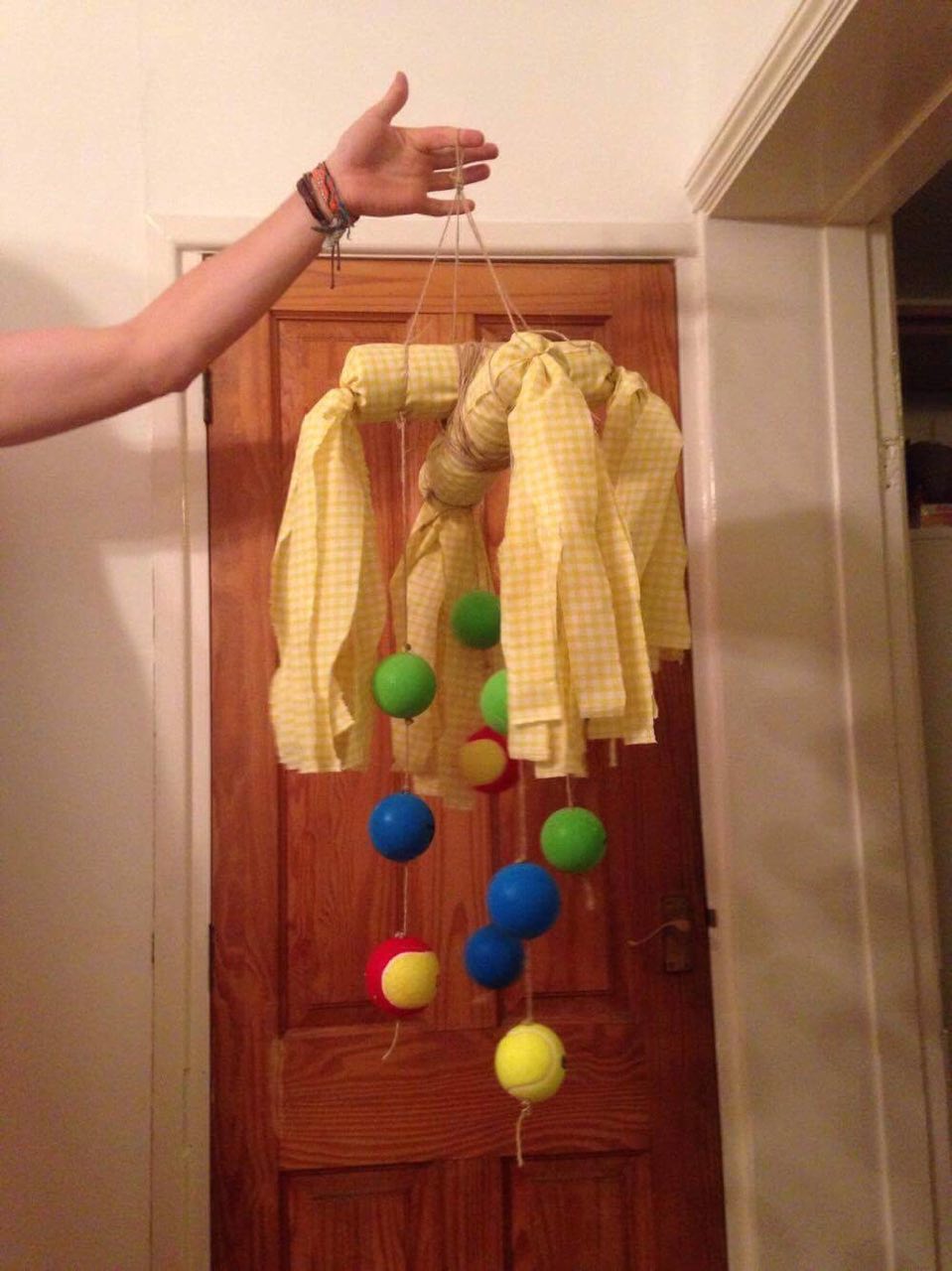


a


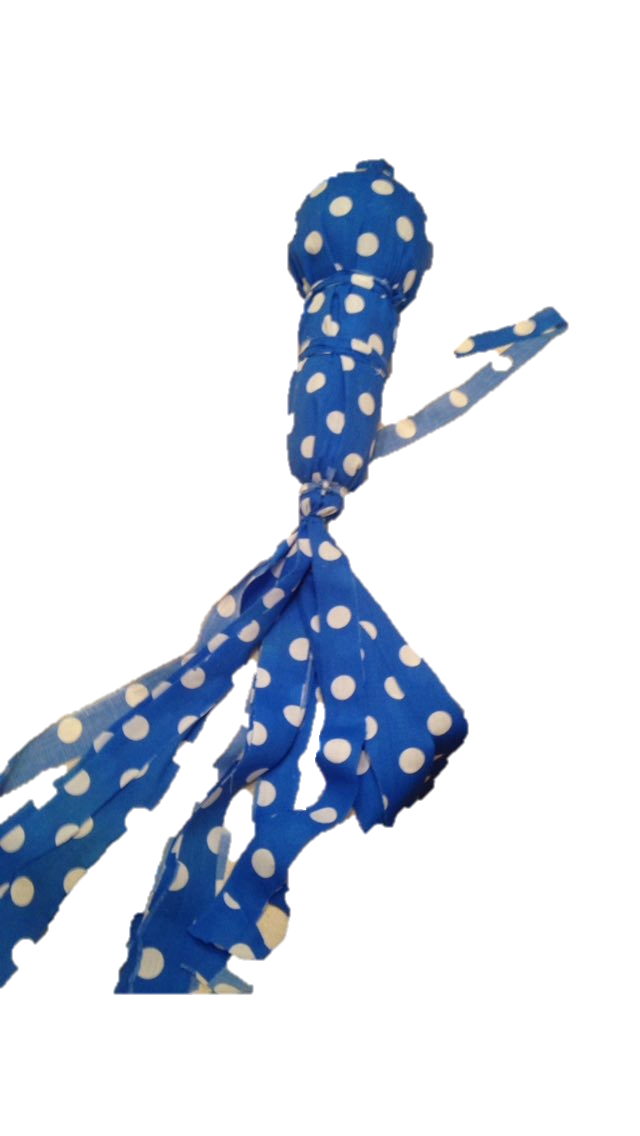


c

b

Supplementary Figure 1: (a-c) Novel objects used in the experiment. Type of object presented to each individual horse was randomized.
